# The prevalence of axial spondyloarthritis in the UK: a cross-sectional cohort study

**DOI:** 10.1186/s12891-015-0853-2

**Published:** 2015-12-21

**Authors:** Louise Hamilton, Alex Macgregor, Andoni Toms, Victoria Warmington, Edward Pinch, Karl Gaffney

**Affiliations:** Rheumatology Department, Norfolk and Norwich University Hospital, Colney Lane, Norwich, Norfolk NR4 7UY UK; Norwich Medical School, University of East Anglia, Norwich, UK; Radiology Department, Norfolk and Norwich University Hospital, Norwich, UK; Humbleyard Surgery, Norwich, UK

**Keywords:** Epidemiology, Ankylosing spondylitis, Questionnaires, Imaging, Spine, Low back pain

## Abstract

**Background:**

Accurate prevalence data are important when interpreting diagnostic tests and planning for the health needs of a population, yet no such data exist for axial spondyloarthritis (axSpA) in the UK. In this cross-sectional cohort study we aimed to estimate the prevalence of axSpA in a UK primary care population.

**Methods:**

A validated self-completed questionnaire was used to screen primary care patients with low back pain for inflammatory back pain (IBP). Patients with a verifiable pre-existing diagnosis of axSpA were included as positive cases. All other patients meeting the Assessment of SpondyloArthritis international Society (ASAS) IBP criteria were invited to undergo further assessment including MRI scanning, allowing classification according to the European Spondyloarthropathy Study Group (ESSG) and ASAS axSpA criteria, and the modified New York (mNY) criteria for ankylosing spondylitis (AS).

**Results:**

Of 978 questionnaires sent to potential participants 505 were returned (response rate 51.6 %). Six subjects had a prior diagnosis of axSpA, 4 of whom met mNY criteria. Thirty eight of 75 subjects meeting ASAS IBP criteria attended review (mean age 53.5 years, 37 % male). The number of subjects satisfying classification criteria was 23 for ESSG, 3 for ASAS (2 clinical, 1 radiological) and 1 for mNY criteria. This equates to a prevalence of 5.3 % (95 % CI 4.0, 6.8) using ESSG, 1.3 % (95 % CI 0.8, 2.3) using ASAS, 0.66 % (95 % CI 0.28, 1.3) using mNY criteria in chronic back pain patients, and 1.2 % (95 % CI 0.9, 1.4) using ESSG, 0.3 % (95 % CI 0.13, 0.48) using ASAS, 0.15 % (95 % CI 0.02, 0.27) using mNY criteria in the general adult primary care population.

**Conclusions:**

These are the first prevalence estimates for axSpA in the UK, and will be of importance in planning for the future healthcare needs of this population.

**Trial registration:**

Current Controlled Trials ISRCTN76873217

## Background

Accurate estimates of disease prevalence are important when planning for future health needs, yet no contemporary data exist for axial spondyloarthritis (axSpA) or ankylosing spondylitis (AS) in the UK. The only general population studies date from the late 1950s, several years before the first AS classification criteria (the Rome criteria) were adopted. [1, 2] In Underwood and Dawes' 1995 study 313 patients with chronic back pain in primary care were screened for inflammatory back pain, with those scoring positively then examined clinically and radiologically. [3] Two patients had AS and 18 (5 %) had at least one feature associated with SpA, but no estimates of prevalence in the general population could be made.

Understanding of the spectrum of axSpA/AS has changed considerably in the past decade, with MRI changes encapsulated into the Assessment of Spondyloarthritis International Society (ASAS) classification criteria. [4] However, to date no European studies have estimated disease prevalence using MRI data. In this study we investigated the prevalence of axSpA in a UK primary care cohort by applying contemporary classification criteria and imaging modalities to patients with inflammatory back pain (IBP) - the earliest and commonest symptom of axSpA. [5]

## Methods

The setting was a large general practice in Norfolk, UK, with 17,000 patients at the time of the study. Potential participants were patients aged 18–80 who had at any time consulted their general practitioner with low back pain. Patients were identified through a READ code search of electronic health records and were excluded from the study if they lived in a nursing home or had a terminal illness. A validated screening questionnaire for axSpA [6] was sent to a random sample of those eligible (all patients whose surnames began with A-G or J). Potential participants were selected pragmatically on the basis of first letter of surname, as the surgery computer system was not configured to allow stratification by other means such as age decile or postcode. A further questionnaire was sent to non-responders.

Respondents with a pre-existing diagnosis of axSpA or AS (verified from hospital or GP records) were included as positive cases. All others whose questionnaire responses indicated they met the ASAS IBP classification criteria were invited for clinical review. This included a detailed history and physical examination by a rheumatologist, and blood testing for HLA-B27. All subjects were invited to undergo an MRI scan of the whole spine and sacroiliac joints, according to the Spondyloarthritis Research Consortium Canada (SPARCC) protocol (http://www.arthritisdoctor.ca/mri.php). Scans were reported by an experienced musculoskeletal radiologist blinded to the clinical data. Subjects were classified according to the European Spondyloarthropathy Study Group (ESSG) SpA criteria [7], the ASAS axSpA criteria (clinical and radiological arms) [4] and the modified New York (mNY) criteria for AS [8] (Table 1).

**Table 1:** SpA classification criteria

ESSG	ASAS	mNY criteria
Inflammatory spinal pain OR synovitis (asymmetric or predominantly lower limb)	Sacroiliitis on imaging plus ≥ 1 SpA feature OR HLA-B27 plus ≥ 2 other SpA features	Definite AS if radiological criterion is associated with at least one clinical criterion.
AND one or more of the following:		Clinical criteria:
• Positive family history	SpA features:	• Low back pain and stiffness for > 3 months that improves with exercise but is not relieved by rest.
• Psoriasis	Inflammatory back pain,	
• Inflammatory bowel disease	arthritis, enthesitis (heel), uveitis, dactylitis,	
• Urethritis, cervicitis or acute diarrhoea within 1 month before arthritis	psoriasis, Crohns/colitis, good response to NSAIDs, family history for SpA, HLA-B27, elevated CRP	• Limitation of motion of the lumbar spine in the sagittal and frontal planes.
• Buttock pain alternating between right and left gluteal areas		• Limitation of chest expansion relative to normal values correlated for age and sex.
• Enthesopathy • Sacroiliitis	Sacroiliitis on imaging:	
	Definite radiographic sacroiliitis according to mNY criteria.	Radiological criterion:
	OR	
	Active (acute) inflammation on MRI highly suggestive of sacroiliitis associated with SpA.	• Sacroiliitis grade ≥ 2 bilaterally or 3–4 unilaterally.

The sample size calculation for the initial survey assumed the prevalence of axSpA in chronic back pain patients to be 3–7 %, with a confidence level of 95 %. The recruitment target was 457 but as the response rate to a postal survey would not be expected to exceed 50 % we aimed to approach around 1000 potential participants.

Ethical approval for the study was granted by the Norfolk Research Ethics Committee, and written consent was obtained from participants prior to clinical review. Data were analysed using PASW 18 and Microsoft Excel. Confidence intervals for the population prevalence estimates were obtained using bootstrapping in Stata (version 12).

## Results

**Fig. 1:**
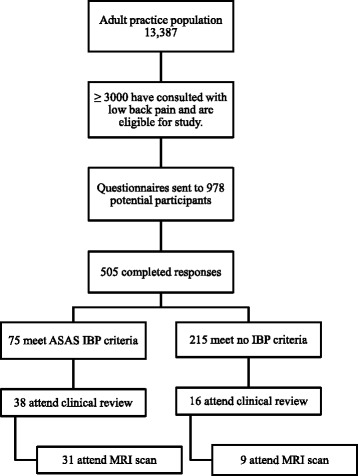
Recruitment of participants at each stage of the study

Recruitment through each stage of the study is shown in Fig. 1. The initial search found over 3000 individuals who had consulted on at least one occasion with low back pain. This represented 22.4 % of the total adult practice population. From this pool of potential participants, questionnaires were sent to a random sample of 978 people with 505 questionnaires returned (response rate 51.6 %). The prevalence of IBP in this cohort has been reported previously [9]. Respondents were significantly older than all potential participants (mean age (95 % CI) 57.5 years (56.4, 58.6) versus 53.2 years (52.2, 54.1) but there was no gender difference (*Χ*^2^*p* = 0.27). Despite being selected on the basis of a single consultation, 80 % of respondents reported pain lasting for at least 3 months.

Six respondents had a pre-existing diagnosis of axSpA (one woman and 3 men met mNY criteria; 2 women met ASAS axSpA criteria, 1 through the clinical arm and one the radiological arm). Of 75 additional subjects who met the ASAS IBP criteria in their questionnaire responses, 38 attended a clinical review appointment. The mean age of clinical review participants was 53.5 years, and 37 % were male. All were white British, with 2 (5 %) positive for HLA-B27. There was no significant difference in age or gender between those who agreed to attend a clinical assessment and the 75 invited (*t*-test *p* = 0.443 and *Χ*^2^ test *p* = 0.888 respectively).

MRI was undertaken in the 31 subjects who consented and had no contra-indication. The prevalence of bone marrow oedema on MRI was low, with clinically significant inflammatory change seen in only one subject (3 %). Despite having typical symptoms meeting the ASAS IBP criteria, 14 (45 %) had only degenerative changes on MRI and 11 (35 %) had a normal scan.

Fifteen respondents self-reported a diagnosis of AS, but on review of primary care and hospital records had other conditions entirely, including spondylosis, osteopenia and polymyalgia rheumatica. As a control, 16 respondents with ‘mechanical’ back pain (not meeting any of the IBP criteria) were assessed, one of whom fulfilled ASAS axSpA criteria through the clinical arm. However, this subject was felt to meet the ASAS IBP classification criteria when his symptoms were reviewed face-to-face. The mean Bath Ankylosing Spondylitis Activity Index (BASDAI) in the 38 subjects with IBP was 3.89, versus 4.91 in the 16 controls (*p* = 0.133), with a mean Ankylosing Spondylitis Quality of Life (ASQoL) score of 4.92 in the subjects versus 6.75 in the controls (*p* = 0.269).

### Prevalence of axSpA

#### ESSG

Following clinical review, 23 of the 38 attendees met the ESSG classification criteria (see Table 2). There was no significant gender difference amongst those fulfilling the criteria (*Χ*^2^ test *p* = 0.503). Despite the mean age of participants, 21/23 (91 %) had onset of back pain by 45 years. Assuming the attendees to be representative, 45 of the 75 respondents identified with IBP would be expected to meet ESSG criteria. Including the six individuals with a pre-existing diagnosis, this equates to a minimum prevalence of ESSG-defined SpA of 5.3 % (95 % CI 4.0, 6.8) among adults aged 18–80 with chronic low back pain. Extrapolating to the adult practice population, the minimum prevalence of ESSG-defined SpA can be estimated at 1.2 % (95 % CI 0.9, 1.4).

**Table 2:** Number of individuals meeting axSpA criteria stratified by age and gender

Age band	Subjects sent screening questionnaire		ASAS IBP + ve		Subjects seen for clinical review		ESSG + ve		ASAS + ve		mNY criteria + ve	
	Male	Female	Male	Female	Male	Female	Male	Female	Male	Female	Male	Female
20–29	27 (2.8 %)	44 (4.5 %)	1 (1 %)	1 (1 %)	0 (0 %)	1 (3 %)	0 (0 %)	1 (4 %)	0 (0 %)	0 (0 %)	0 (0 %)	0 (0 %)
30–39	58 (5.9 %)	67 (6.8 %)	5 (7 %)	8 (11 %)	2 (5 %)	3 (8 %)	0 (0 %)	2 (9 %)	0 (0 %)	0 (0 %)	0 (0 %)	0 (0 %)
40–49	94 (9.6 %)	98 (10.0 %)	4 (5 %)	12 (16 %)	3 (8 %)	7 (18 %)	1 (4 %)	4 (17 %)	1(33 %)	0 (0 %)	0 (0 %)	0 (0 %)
50–59	107(10.9 %)	109 (11.1 %)	8 (11 %)	14 (19 %)	3 (8 %)	6 (16 %)	2 (9 %)	5 (22 %)	0 (0 %)	0 (0 %)	0 (0 %)	0 (0 %)
60–69	116 (11.9 %)	114 (11.7 %)	6 (8 %)	9 (12 %)	3 (8 %)	4 (11 %)	2 (9 %)	2 (9 %)	1 (33 %)	0 (0 %)	0 (0 %)	0 (0 %)
70–79	65 (6.6 %)	79 (8.1 %)	4 (5 %)	3 (4 %)	3 (8 %)	3 (8 %)	2 (9 %)	2 (9 %)	1 (33 %)	0 (0 %)	1 (100 %)	0 (0 %)
*Total*	*467*	*511*	*28*	*47*	*14*	*24*	*7*	*16*	*3*	*0*	*1*	*0*

#### ASAS axSpA

Three subjects satisfied the ASAS axSpA criteria (one through the radiological arm and 2 the clinical arm). Including the six respondents with a pre-existing diagnosis this equates to an estimated minimum prevalence of ASAS-defined axSpA of 1.3 % (95 % CI 0.8, 2.3) in adults with chronic back pain and 0.3 % (95 % CI 0.13, 0.48) in the adult primary care population.

#### mNY criteria

One of the three subjects who fulfilled ASAS axSpA criteria also met mNY criteria (radiographs of the sacroiliac joints having been performed outwith the study), as did four respondents with a pre-existing diagnosis. This equates to a minimum AS prevalence of 0.66 % (95 % CI 0.28, 1.3) amongst those with chronic low back pain. Extrapolated to the general practice population, the prevalence is 0.15 % (95 % CI 0.02, 0.27).

## Discussion

This study has estimated the prevalence of axSpA in a primary care population to be 1.2 % using ESSG criteria, 0.3 % using ASAS criteria, and 0.15 % using mNY criteria. These are the first such estimates for axial SpA in the UK, and will be important when planning the provision of health services for these patients. Previous European studies using ESSG criteria have provided similar estimates of 0.3 % [10] – 1.06 % [11], while the prevalence of axSpA meeting ASAS criteria (without MRI imaging) in a recent French cohort was 0.43 % [12].

The mNY criteria – in common with all classification criteria – were developed by characterising patients with definite disease. In axSpA, this means patients at the extreme end of the spectrum with significant radiographic damage, which might only manifest a decade or more after symptom-onset. [13] The ASAS criteria were intended to improve sensitivity in early disease, and as well as the ‘radiographic’ group classify as axSpA those with MRI-defined sacroiliitis and patients who are HLA-B27 positive and have clinical features of axSpA but no definite radiological abnormalities. This ‘clinical arm’ of the criteria has proven controversial, with concerns that patients with other conditions such as fibromyalgia could be misclassified as having axSpA. Furthermore axSpA as a concept doesn’t necessarily equate with ‘early AS’; the proportion of patients with axSpA who eventually meet mNY criteria ranges from 14 to 59 % in the literature. [13, 14] While the ESSG criteria are also better at classifying early disease, their specificity in real-life situations is limited. Of 28 patients in a Spanish study meeting ESSG criteria, only 13 had clinician-confirmed SpA at 5 years [15]. However, most studies of SpA prevalence have used the ESSG criteria to classify cases, and including them in this study allowed direct comparison to be made with previous studies. The ESSG criteria can also be fulfilled by patients with peripheral spondyloarthropathy, so using them alone might over-estimate the prevalence of axial disease in the community. This is less likely to be an issue in our study where back pain was the initial screening question and no patients had peripheral arthritis on review.

The main limitation in the study design is the risk of selection bias at each sampling stage, potentially leading to overestimation of disease prevalence. While the demographic characteristics of respondents and non-respondents were similar, we have no information on the symptom severity of non-attenders. The assumption is that participants would be driven to attend review and MRI appointments by more intrusive symptoms, although this does not necessarily equate to more axial SpA. The majority of patients with IBP had no changes or only degenerative changes on MRI, and patients with mechanical pain had similar patient-reported outcome measure scores to those with inflammatory-type pain.

The population prevalence estimates in this study are minimum values, with two assumptions: that none of the non-responders to the questionnaire had IBP or by extension axSpA, and that the 22.4 % of patients who had consulted with back pain included all the cases of axSpA (diagnosed and undiagnosed) in the practice. While up to 30 % of patients with physician-diagnosed early axSpA might have non-inflammatory back pain [16], the proportion in this study was much smaller and there is unlikely to have been a significant underestimation of prevalence resulting from the review of patients with IBP rather than chronic back pain per se. Although the absolute numbers in the imaging phase were small, the sample size was sufficient to assess the prevalence of axSpA with reasonable precision, and allowed three subjects with previously unsuspected disease to be classified as having axSpA. This represents one third of those who fulfilled the ASAS axSpA criteria by the end of the study.

Fewer than 10 % of people in this study who met the ASAS IBP criteria fulfilled the ASAS axSpA criteria. This is in contrast to primary care screening studies such as the RADAR [17] and MASTER [18] studies where the proportion with physician-diagnosed SpA was consistently around 40 %. Similarly a recent Dutch study showed 1 in 4 young patients with chronic low back pain met the ASAS axSpA criteria. [19] Referral studies, in which patients meeting pre-specified criteria are triaged for further investigation, tend to exclude those aged over 45 years which may be a factor, although the populations are different in other ways. In the RADAR and MASTER studies primary care physicians were incentivised to refer to specialist centres with an interest in axSpA, and awareness of the condition was already heightened amongst patients and doctors. In contrast our study recruited participants from the general primary care population, few of whom were seeking current medical attention for their pain, and where knowledge of AS was low given the number of respondents who mistakenly believed they had the condition. We believe the 10 % rate of axSpA is likely to better reflect the true frequency among people with inflammatory-type back pain.

## Conclusions

These are the first UK estimates of axSpA prevalence in general practice using contemporary criteria. The findings suggest there is a hidden burden of axSpA and even AS in primary care. Given the excellent treatment outcomes observed for axSpA with new therapies, there is an urgent need to refine clinical assessment tools to detect axSpA specifically for use in the population setting.

## References

[1] Lawrence JS (1963). The prevalence of arthritis. Br J Clin Pract.

[2] Kellgren JH, Lawrence JS, Aitken-Swan J (1953). Rheumatic complaints in an urban population. Ann Rheum Dis.

[3] Underwood MR, Dawes P (1995). Inflammatory back pain in primary care. Br J Rheumatol.

[4] Rudwaleit M, van der Heijde D, Landewé R, Listing J, Akkoc N, Brandt J (2009). The development of Assessment of SpondyloArthritis international Society classification criteria for axial spondyloarthritis (part II): validation and final selection. Ann Rheum Dis.

[5] Rojas-Vargas M, Muñoz-Gomariz E, Escudero A, Font P, Zarco P, Almodovar R (2009). First signs and symptoms of spondyloarthritis--data from an inception cohort with a disease course of two years or less (REGISPONSER-Early). Rheumatology (Oxford).

[6] Hamilton L, Macgregor A, Newman D, Belkhiri A, Toms A, Gaffney K (2013). Validation of a patient self-reported screening questionnaire for axial spondyloarthropathy in a UK Population. Spine (Phila Pa 1976).

[7] Dougados M, van der Linden S, Juhlin R, Huitfeldt B, Amor B, Calin A (1991). The European spondylarthropathy study group preliminary criteria for the classification of spondylarthropathy. Arthritis Rheum.

[8] Van der Linden S, Valkenburg HA, Cats A (1984). Evaluation of diagnostic criteria for ankylosing spondylitis. A proposal for modification of the New York criteria. Arthritis Rheum.

[9] Hamilton L, Macgregor A, Warmington V, Pinch E, Gaffney K (2014). The prevalence of inflammatory back pain in a UK primary care population. Rheumatology (Oxford).

[10] Roux CH, Saraux A, Le Bihan E, Fardellone P, Guggenbuhl P, Fautrel B (2007). Rheumatoid arthritis and spondyloarthropathies: geographical variations in prevalence in France. J Rheumatol.

[11] De Angelis R, Salaffi F, Grassi W (2007). Prevalence of spondyloarthropathies in an Italian population sample: a regional community-based study. Scand J Rheumatol.

[12] Costantino F, Talpin A, Said-Nahal R, Goldberg M, Henny J, Chiocchia G (2013). Prevalence of spondyloarthritis in reference to HLA-B27 in the French population: results of the GAZEL cohort. Ann Rheum Dis.

[13] Mau W, Zeidler H, Mau R, Majewski A, Freyschmidt J, Stangel W (1990). Evaluation of early diagnostic criteria for ankylosing spondylitis in a 10 year follow-up. Z Rheumatol.

[14] Aydin SZ, Maksymowych WP, Bennett AN, McGonagle D, Emery P, Marzo-Ortega H (2011). Validation of the ASAS criteria and definition of a positive MRI of the sacroiliac joint in an inception cohort of axial spondyloarthritis followed up for 8 years. Ann Rheum Dis.

[15] Collantes E, Veroz R, Escudero A, Muñoz E, Muñoz MC, Cisnal A (2000). Can some cases of “possible” spondyloarthropathy be classified as “definite” or “undifferentiated” spondyloarthropathy? Value of criteria for spondyloarthropathies. Spanish Spondyloarthropathy Study Group. Joint Bone Spine.

[16] Tomero E, Mulero J, de Miguel E, Fernández-Espartero C, Gobbo M, Descalzo MA (2014). Performance of the Assessment of Spondyloarthritis International Society criteria for the classification of spondyloarthritis in early spondyloarthritis clinics participating in the ESPERANZA programme. Rheumatology (Oxford).

[17] Sieper J, Srinivasan S, Zamani O, Mielants H, Choquette D, Pavelka K (2013). Comparison of two referral strategies for diagnosis of axial spondyloarthritis: the Recognising and Diagnosing Ankylosing Spondylitis Reliably (RADAR) study. Ann Rheum Dis.

[18] Poddubnyy D, Vahldiek J, Spiller I, Buss B, Listing J, Rudwaleit M (2011). Evaluation of 2 screening strategies for early identification of patients with axial spondyloarthritis in primary care. J Rheumatol.

[19] Van Hoeven L, Luime J, Han H, Vergouwe Y, Weel A (2014). Identifying axial spondyloarthritis in Dutch primary care patients, ages 20–45 years, with chronic low back pain. Arthritis Care Res (Hoboken).

